# Macromolecular protein crystallisation with biotemplate of live cells

**DOI:** 10.1038/s41598-022-06999-7

**Published:** 2022-02-22

**Authors:** Mubai Sun, Huaiyu Yang, Xinyu Miao, Weixian Wang, Jinghui Wang

**Affiliations:** 1 Agricultural Products Processing Research Institute, Jilin Academy of Agricultural Science, Changchun, 130124 Jilin China; 2 Department of Chemical Engineering, Loughborough University, Leicestershire, LE113TU UK; 3 School of Materials and Chemistry, University of Shanghai for Science and Technology, Shanghai, 200093 China

**Keywords:** Biotechnology, Microbiology, Chemical engineering, Biomaterials - cells

## Abstract

Macromolecular protein crystallisation was one of the potential tools to accelerate the biomanufacturing of biopharmaceuticals. In this work, it was the first time to investigate the roles of biotemplates, Saccharomyces cerevisiae live cells, in the crystallisation processes of lysozyme, with different concentrations from 20 to 2.5 mg/mL lysozyme and different concentrations from 0 to 5.0 × 10^7^ (cfu/mL) Saccharomyces cerevisiae cells, during a period of 96 h. During the crystallisation period, the nucleation possibility in droplets, crystal numbers, and cell growth and cell density were observed and analysed. The results indicated the strong interaction between the lysozyme molecules and the cell wall of the S. cerevisiae, proved by the crystallization of lysozyme with fluorescent labels. The biotemplates demonstrated positive influence or negative influence on the nucleation, i.e. shorter or longer induction time, dependent on the concentrations of the lysozyme and the S. cerevisiae cells, and ratios between them. In the biomanufacturing process, target proteins were various cells were commonly mixed with various cells, and this work provides novel insights of new design and application of live cells as biotemplates for purification of macromolecules.

## Introduction

Therapeutic proteins provide effective treatments to a wide range of medical conditions and their demands have grown rapidly over the past two decades. The global sale of mAb products almost doubled from 2008 to 2013, reaching nearly $75 billion, and is expected to increase faster in this decade^1^. The purification of therapeutic proteins is very expensive, due to the high cost of the chromatography method^2^ for recovering the resin and scaling up^3,4^, as well as a large footprint for the product and environment^5,6^. Crystallisation has been a potential purification and isolation method^7–11^, which widely used in small organic molecular pharmaceutical purification. Macromolecular protein crystallisation^12^ has the potential to significantly simplify the downstream biomanufacturing process, achieving a significant waste reduction. Therapeutic proteins in the crystalline form have multiple advantages over their solution counterpart including higher stability^13–15^. However, due to the difficulty of large and flexible proteins forming highly ordered structure^16,17^, nucleation and crystal growth are still challenging in industrial applications. More research on protein crystallisation was reported and with attempts to accelerate the nucleation and as the purification process is limited by the crystallisation time^18^. Different solid templates (i.e. heterogeneous particles and solid surfaces) have been investigated to improve the protein crystallisation efficiency^19–26^ and shorten the crystallisation process. Protein crystallisation becomes more difficult due to the presence of biological impurities such as cell debris, host cell protein, DNA and virus, among which host cell protein normally has the highest concentration and hence poses the greatest hindrance to the success of crystallisation^27^. In order to better connect the upstream bioprocess and downstream bioprocess, it is critical to understand the influence of the impurities such as cells on the crystallisation process.

Saccharomyces cerevisiae (S. cerevisiae) with a short growth cycle and strong fermentation capacity, is widely used in the large-scale production of wine, foods, chemical products and pharmaceuticals^28–30^. Its diameter is 5–10 μm, and the cells are spherical or oval. The cell wall of yeast, for maintaining cell morphology and intercellular recognition, including 30% dry weight of mannan, 30% of β-Dextran, 20% of glycoprotein and chitin, and others^31^. Mannan has a three-dimensional spiral structure in space, and it also has a variety of physiological functions such as cell recognition and control of cell wall pore size. Mannan is chemically stable, with a relatively stable pH between 2.5 and 8.0^32,33^. Lysozyme can act on the β-1,4 bond between N-acetylmuramic acid and N-acetylglucosamine in the peptide polysaccharide molecule, which destroys the cell wall of bacteria, losing its protective effect on cells, and finally makes bacteria dissolve and die^34^. However, lysozyme has limited impact on the cell wall lysis of the S. cerevisiae, due to the lack of lysozyme sites in its cell wall^35,36^.

To the best knowledge of the authors, the current study is the first to apply the biotemplates, live-cell templates, on protein crystallisation, this research will fill the technology gap by demonstrating the feasibility of yeast cells, S. cerevisiae, as a heterogeneous nucleant to promote the crystallisation of a model protein, lysozyme. The work also demonstrated the crystallisation technology has the ability to isolate protein in the complex solution environment with various concentrations of the live cells. The knowledge provided in this study can be transformed into future applications of biotemplate design for bioseparation and purification of therapeutic proteins or other macromolecules.

## Results

**Figure 1:**
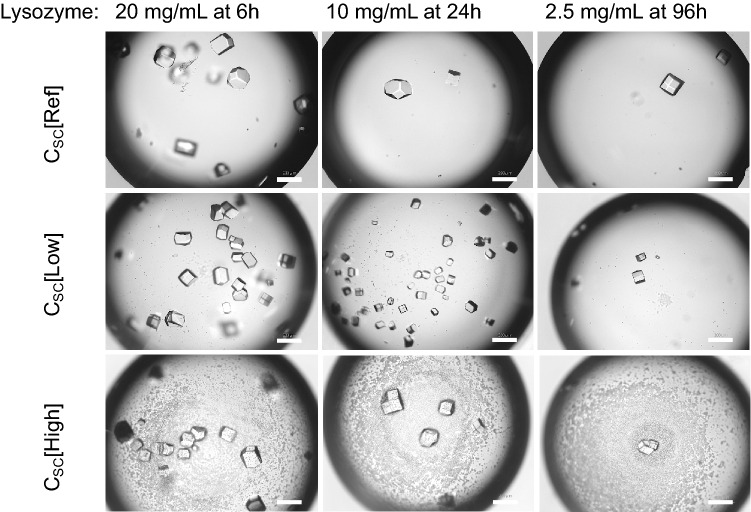
Microscope images of some droplets with the C_Lys_ of 20, 10 and 2.5 mg/mL and with three different concentrations of biotemplates. Scale bars are 200 μm.

Figure 1 shows the examples of formation of protein crystals inside droplets with and without biotemplates at various time dependent on the concentration of the protein. The droplets with biotemplates at C_SC_[Low] of the biotemplates had much more crystals, and the crystals were obviously smaller, as well as more uniformed, than the crystals obtained in the droplets with biotemplates at C_SC_[High]. It is obvious that plenty of S. cerevisiae cells were observed in the droplets with biotemplates at C_SC_[High], much fewer S. cerevisiae was observed in the droplets with biotemplates at C_SC_[Low]. The crystals had light agglomerations with a lower concentration of biotemplates, but there was no agglomeration observed in the droplets without and with a high concentration of S. cerevisiae. There was no obvious trend in the change of the crystal shape in the droplets with different concentrations of the biotemplates.

**Figure 2:**
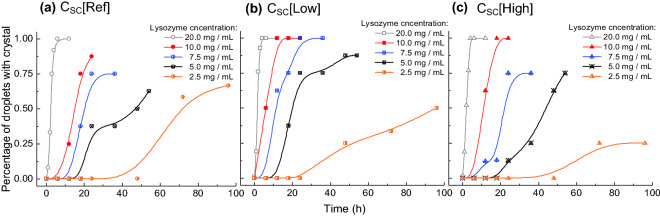
Percentage of droplets with crystals during crystallisation time of 96 h in the solution of 2.5–20.0 mg/mL lysozyme and without biotemplates (**a**), with a low concentration of biotemplates (**b**) and high concentration of biotemplates (**c**).

**Figure 3:**
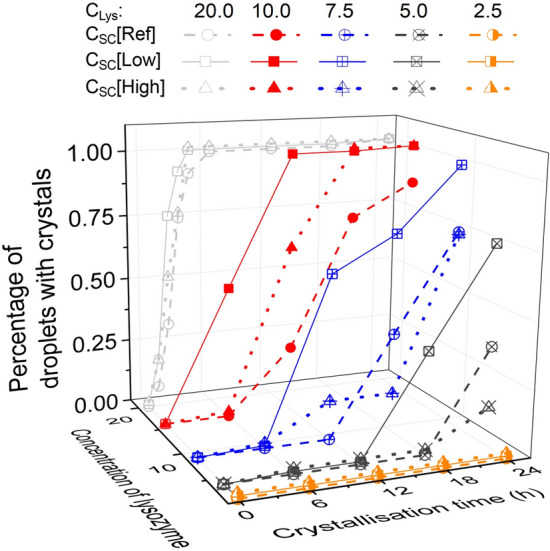
Percentage of droplets with crystals during crystallisation time of 24 h in the solutions with 2.5–20.0 mg/mL lysozyme, and without and with a low and high concentration of biotemplates.

Figure 2 shows the accumulative possibility of the nucleation, i.e. the percentage of droplets with at least one crystal inside. The longer crystallisation time was, the nucleation occurred in more droplets at all the conditions. With the same concentration of S. cerevisiae biotemplates in the solution, the crystallisation time decreased with an increase in the concentration of lysozyme. The nucleation percentage curves show consistent trends, as the higher concentration of the lysozyme in the solution was, the higher driving force (supersaturation) for the nucleation in the droplets could become. When the C_Lys_ was 2.5 mg/mL, the nucleation time increased dramatically, only 20% of droplets nucleated in 60 h crystallisation time with or without biotemplates. When the C_Lys_ increased to 7.5 mg/mL and above, nucleation occurred in more than 50% of droplets in 20 h, and there was nucleation in less than 4 h in all the droplets with the C_Lys_ of 20 mg/mL. However, it is noted that with different concentrations of biotemplates, the crystallisation percentages were different in the droplet with the same C_Lys_. Figure 3 shows the percentage change of the droplets with nucleation in 0–24 h crystallisation time. When the C_Lys_ was 2.5 mg/mL, no nucleation was observed in 24 h, and with an increase in the C_Lys_, more droplets with crystals were observed, and there were crystals in almost all of the droplets with C_Lys_ above 7.5 mg/mL at 24 h.

**Figure 4:**
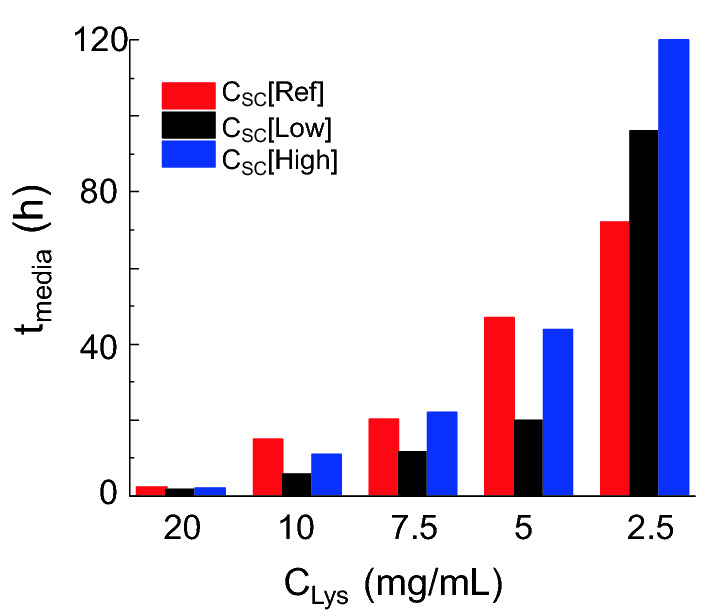
Median induction time extrapolated from Fig. 2 in the droplets with the C_Lys_ of 2.5–20 mg/mL and three different concentrations of the biotemplates.

It is obvious that the S. cerevisiae as biotemplate had strong influences on the nucleation and crystallisation process, and positive or negative influences were dependent on the concentrations of the lysozyme and the biotemplates. The possibility of the nucleation at 24 h follow the order in the droplets at an equal C_Lys_ of 10 or 20 mg/mL: with C_SC_[Low] > with C_SC_[High] > with C_SC_[Ref], and at an equal C_Lys_ of 5 or 7.5 mg/mL, the order changed to: with C_SC_[Low] > with C_SC_[Ref] > with C_SC_[High]. At 2.5 mg/mL, the order of the nucleation possibility in the droplets became: with C_SC_[Ref] > with C_SC_[Low] > with C_SC_[High], shown in Fig. 2. At the high C_Lys_, S. cerevisiae as biotemplates increased the nucleation rate, at the middle range of the C_Lys_, only a low concentration of S. cerevisiae prompted the nucleation, and the high concentration of S. cerevisiae hindered the nucleation compared with the droplets without S. cerevisiae. The trend was consistent that with the C_Lys_ decreased to 2.5 mg/mL, both high and low concentrations of S. cerevisiae slowed the nucleation rates. The trend indicates that the biotemplates would accelerate the nucleation possibility, but too many templates would lead to an opposite effect. The results were also in the agreement with the median induction time shown in Fig. 4. The induction times were all shorter with biotemplates in the droplets, except in the condition with the C_Lys_ at 2.5 mg/mL. The high concentration of the biotemplates tended to be of less effect or negative effect in the droplets with lower C_Lys_. The overall median induction time increased with a decrease in the C_Lys_ as expected.

**Figure 5:**
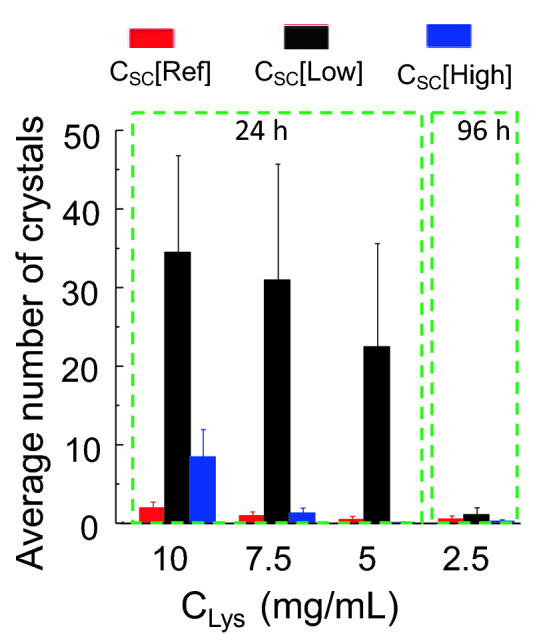
Average numbers of crystals obtained in each droplet at the C_Lys_ of 10, 7.5 and 5 mg/mL at 24 h, and at C_Lys_ of 2.5 mg/mL at 96 h.

The low concentration of biotemplates did not only facilitate the nucleation, but also boosted the quantity of the crystals in the droplets. Due to different time scales for different crystallisation conditions, three groups at different C_Lys_ were compared. At C_Lys_ of 20 mg/mL, the average number of crystals in each droplet were all over one hundred in less than 8 h, and the differences between the droplets with different concentrations of biotemplates were not obviously observed. Figure 4 shows the other two groups. In the lysozyme concentration range of 10–5 mg/mL, at 24 h the average number without biotemplates and with a high concentration of template were all below 10, but the number of crystals with a low concentration of the bio-templates were much higher, with above 8 times differences. At very low C_Lys_, there was few droplets with crystals at 24 h, and at 96 h, there were only a few crystals in each droplet without big differences for droplets with different concentrations of biotemplates. In all the conditions, the average numbers of crystals in the droplets with biotemplates at C_SC_[Low] were much higher than the droplets without biotemplates. The average numbers of crystals in the droplets with biteplates at C_SC_[Low] were higher than those without biotemplates, but the order tended to be reversed in the droplets with low C_Lys_ (Fig. 5).

**Figure 6:**
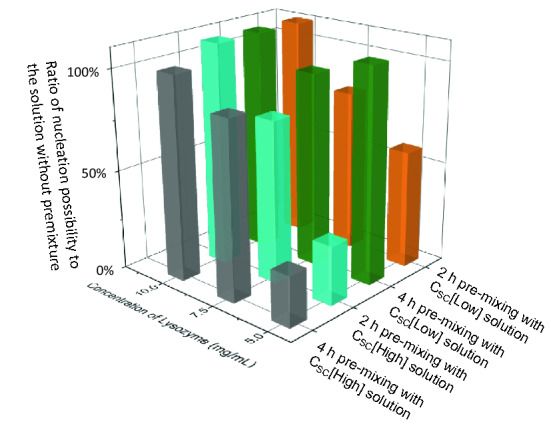
Ratios of nucleation possibility between the droplets with C_Lys_ at 5–10 mg/mL and with premixed bio-templates at C_SC_[Low] or C_SC_[High] for 2 h and 4 h to the droplets with equal condition (no premixing).

To further understand the interactions of the biotemplates with lysozyme protein, the solutions with S. cerevisiae at C_SC_[Low] or C_SC_[High] were pre-mixed with the protein solution of the C_Lys_ of 5–10 mg/mL for 2 and 4 h before adding precipitation solution into the droplet. The percentage of droplets with crystals inside were compared, shown in Fig. 6 at 12 h after mixing with precipitation solution. The droplets with protein solution premixed with bio-templates had a slower nucleation rate than the droplets without premixing of the biotemplates in most of the cases. The stronger negative influence on nucleation was observed in the droplets with low C_Lys_, and in the droplets with C_Lys_ of 5.0 mg/mL and with biotemplates at C_SC_[High], only less than 70% of droplets occurred nucleation compared with the droplets without premixing of the bio-templates at equal crystallisation condition. In the droplets with C_Lys_ of 7.5 mg/mL and with the bio-templates premixed, overall less than 20% of the droplets occurred nucleation compared with the droplets without the premixing of the biotemplates at equal crystallisation condition. There was no obvious influence of the pre-mixing of the biotemplates in the droplets of C_Lys_ at 10 mg/mL, and the premixed biotemplates at C_SC_[High] tended to have stronger negative influence than those at C_SC_[Low]. There was no obvious trend in comparison between the droplets with the premixed biotemplates for 2 h and 4 h, indicating the interactions between the lysozyme and the biotemplates were established in a short period.

**Figure 7:**
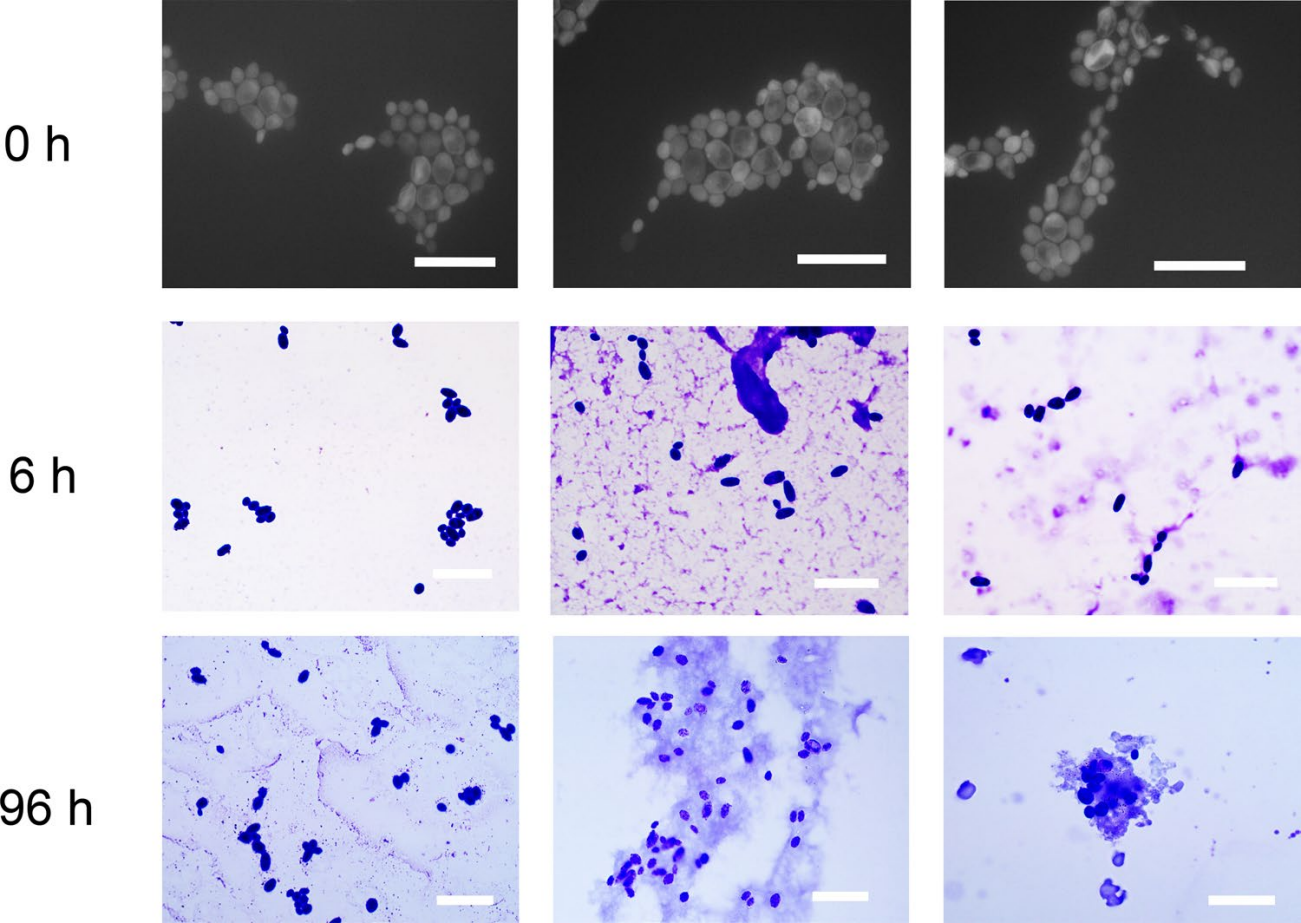
Cell culture of S. cerevisiae in the buffer solution, the lysozyme solution and the crystallisation solution (protein solution mixed with precipitation solution) from left to right columns, respectively, with scale bar of 20 μm.

**Figure 8:**
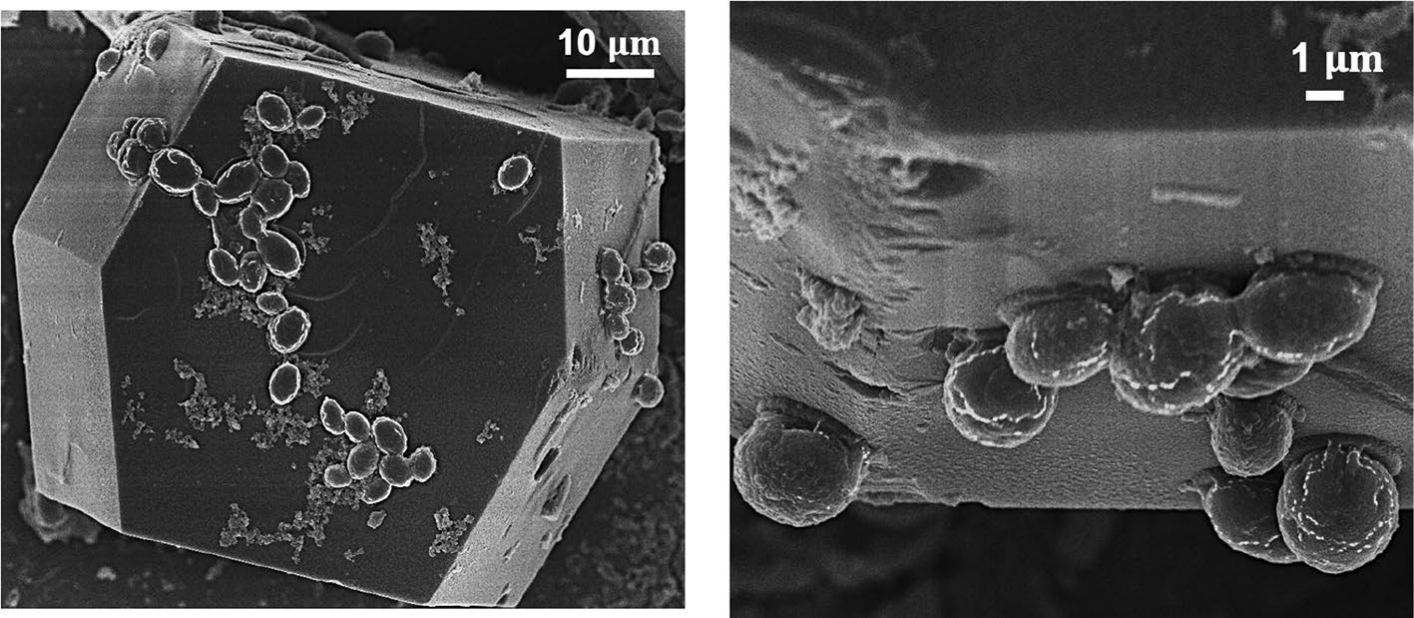
SEM images of S. cerevisiae and lysozyme crystal from hanging-drop experiments with C_SC_[Low] biotemplates and 20 mg/mL lysozyme.

Figure 7 shows that there was no obvious change in the morphology of the S. cerevisiae in the buffer solution, the lysozyme solution or the crystallisation solution (lysozyme solution mixed with sodium chloride solution). Under the fluorescence microscope, there was no obvious fragmentation of the S. cerevisiae observed during 96 h in two kinds of solutions with the lysozyme, indicating that lysozyme did not lead to the lysis of the S. cerevisiae, which was in agreement with the literatures^35,36^. Most S. cerevisiae survived with lysozyme in the solution, and maintained its morphology during the crystallisation, shown in SEM images of Fig. 8. It was reported that S. cerevisiae is capable to overcome stress and survive with good tolerance to ethanol, high salt concentrations and oxidative damage^37,38^.

The results of the spread plate method in Table 1 show that the density of S. cerevisiae cells in the three solutions all increased, but increase rates were much slower than the growth rate in culture solutions with suitable conditions. It was reported that in a suitable concentration of glucose, yeast cells grew exponentially by fermentation and the number of cells would be double in 90 min^39^, leading to a 16 folds increase in 6 h. In this work, at 6 h, S. cerevisiae increased only 4–5 folds in the solution without lysozyme and increased less than 2 folds in two solutions with lysozyme. At 96 h, the densities of S. cerevisiae all further increased, about 7 folds and 4–5 folds in the solutions with lysozyme and without lysozyme, respectively. It is noticed that with lysozyme the density of S. cerevisiae was much lower than the solution without lysozyme, showing the interactions between the lysozyme and the cells, and in the solution with lysozyme without salt was slightly higher than the solution with lysozyme and salt.

**Table 1:** The concentrations of S. cerevisiae in the buffer solution, the lysozyme solution and the crystallisation solution (lysozyme with salt).

Solution	Concentration of S. cerevisiae (10^7^ cfu/mL)		
	In buffer solution	In lysozyme	In lysozyme + salt
0 h	1.0	1.0	1.0
6 h	4.5	1.6	1.4
96 h	6.9	4.6	4.4

## Discussion

**Figure 9:**
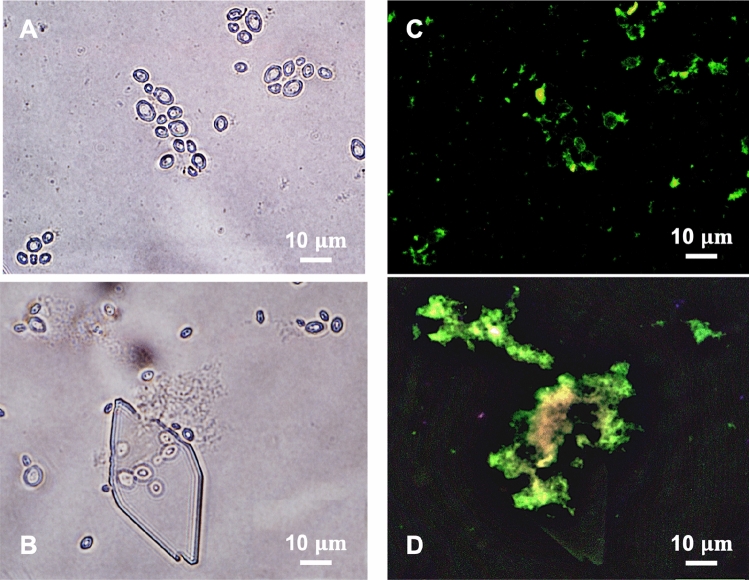
Optical microscope images of the fluorescent lysozyme before nucleation (**A**) and after nucleation (**B**), and corresponding fluorescence microscope images (**B**) and (**D**).

The interactions between the surface/cell wall of S. cerevisiae with the lysozyme affected the crystallisation processes. As heterogeneous surfaces would accelerate the nucleation by decreasing the nucleation free energy^40,41^. Compositions of cell wall of the S. cerevisiae, such as mannoproteins, polysaccharide complexes, and glucan network could be possible heterogeneous nucleants for the lysozyme crystallisation. Some nanoparticles can be encapsulated inside lysozyme crystal^42,43^, but it is not possible to trap S. cerevisiae due to its big size, and, therefore, all the biotemplates should be outside the crystal or on the surface of the crystal, shown in Fig. 8. The observation of the crystallisation process, the cell culture process (Fig. 7), the cell density (Table 1) and the SEM images (Fig. 8) supported that the lysozyme did not lead to the lysis of the S. cerevisiae cells, but obviously hindered the growth and proliferation of the cells, indicating strong interactions between the cell wall and the lysozyme molecules. These interactions were also proved by the images of the crystallisation of the fluorescent lysozyme in Fig. 9. At the initial stage, after mixing the lysozyme with biotemplates, the fluorescent lysozyme fast attached on the cells, making all cells fluorescent, and there were also aggregations of lysozyme around cells, shown in Fig. 9C. At later stage, during and after nucleation, more aggregations were observed around the cells. The interactions between protein and protein were reported in other systems that aggregations of fluorescent-tag proteins were tracked under the fluorescence microscope^44,45^. The adsorption on the cell surface or the aggregation can result from electrostatic interactions^44,46,47^, as one of most factors to influence the folding and binding of the protein, and the rate of protein–protein association, which also supported by the simulation with the docking methods^48^. Therefore, these interactions could result in adsorption or accumulation of lysozyme molecules on the cell surface, contributing to the promotion of the aggregation process during the pre-nucleation process and crystal growth process. Similar phenomena were reported in other systems, such as the formation of crystals of chernikovite salts on the surface of S. cerevisiae^49^, and biosorption of copper^50^ ions on the cell surface of S. cerevisiae. It is noted that only edge of the crystals of the tagged lysozyme appears fluorescent in Fig. 9 (see crystals in the whole droplet in supporting information), indicating the crystalline lysozyme disabled the fluorescent tag but the lysozyme starting to grow on the crystal surface still maintain fluorescent.

Most of the crystallisation experiments were completed at 96 h, during which period the density of S. cerevisiae increased less than 5 folds. The increase in the number of the biotemplates would influence heterogeneous nucleation, due to an increase in heterogeneous surface areas in the crystallisation solution. This can be one of the explanations of the trend shown in Fig. 6, higher nucleation possibility in the droplets with biotemplates premixed in the lysozyme solution. However, compared with two concentrations of the biotemplates between C_SC_[Low] and C_SC_[High] (which was of 100 times difference), the influences of changes in S. cerevisiae cell number were very limited. Therefore, the differences in induction time and crystals formation in the droplets, shown in Figs. 4 and 5, were mainly resulted from the initial concentration of the biotemplates in the crystallisation solution.

There were positive influences on the nucleation and crystal growth in the crystallisation solution of all the lysozyme concentrations, except 2.5 mg/mL, with biotemplates at both C_SC_[Low] and C_SC_[High]. However, a further large increase in the concentration of the biotemplates (at concentrations from C_SC_[Low] to C_SC_[High]) led less positive influences, i.e. the slower nucleation rates with the biotemplates at C_SC_[High] than with the biotemplates at C_SC_[Low]. Moreover, the ratio between the concentrations of the cells and the lysozyme would also determine the influence on the nucleation process, as negative influences were observed in the crystallisation solution of very low lysozyme concentration at 2.5 mg/mL with biotemplates at both low and high concentrations. The higher ratio between the concentration of the biotemplates to the lysozyme, the weaker the positive influence of biotemplates tended to be, turning into negative influences at very high ratios. It is noticed that the influences on the crystallisation process were not linear correlated to the ratio between concentrations of biotemplates and lysozymes. The possible reason can be that a large number of the S. cerevisiae would attract huge amount of the lysozyme molecules, diluting or hindering the formation of aggregations which could result in relatively low concentrations of lysozyme near or attach on the cell surface of each cell. Therefore, the high ratio between the number of biotemplates to the lysozyme molecules lead to the decrease in the driving force of the nucleation, hindering the crystallisation processes.

## Conclusions

Various protein crystallisation processes with biotemplates of live cells were first time observed, proved to be contributed by the interactions between cell walls and lysozyme. The interactions were observed by the fluorescence lysozyme attached on the cell walls and aggregated around cells. The biotemplates of S. cerevisiae at low concentrations could facilitate the nucleation, with more droplets having crystals inside during the same period, and crystal growth of lysozyme, with the formation of more crystals. With the lysozyme in the solution, the cells would still grow without lysis but the density of the cells only slowly increased, indicating strong interactions between the lysozyme and the cell wall of S. cerevisiae. Protein molecules adhered on the cell wall of S. cerevisiae and aggregated around, and then the cell wall as heterogeneous surface decreased the nucleation free energy, leading to positive influences on the nucleation. These positive influences became weaker with a decrease in the concentration of lysozyme or a large increase in the concentration of biotemplates. With much lower ratios between concentration of the lysozyme to the biotemplates, the nucleation was hindered with a longer induction time. The crystal shape was not obviously influenced by the biotemplates at both high and low concentrations, but with low concentration of the biotemplates, there were always times more crystals formed in the droplets with more uniform size distributions.

## Materials and methods

### Materials

Lysozyme (≥ 90%) was purchased from Sigma Aldrich (Gillingham, UK); FITC-labelled fluorescent lysozyme (> 90%) was purchased in Ruixi Biology); Chinasodium chloride, acetic acid glacial (> 99.7%) was purchased from Beijing Chemical Works (Beijing, China); sodium acetate (> 99.7%) was purchased from Xilong Scientific (Guangdong, China). Surfactant-free cellulose acetate membrane filter (0.22 µm) was purchased from Green Mall (Jiangsu, China). FDA/PI staining kits were purchased from BestBio (Shanghai, China). De-ionised water was used, and all chemicals were used as received without further purification.

### Biotemplates preparation

S. cerevisiae was activated with LB medium, placed in a constant temperature incubator at 20 °C $\pm$ 0.2 °C, and cultured at 140 r/min for 0–96 h. The activated cell culture solution was diluted in sodium chloride solutions with different ccentrations of S. cerevisiae to prepare the precipitation solution used in protein crystallisation. The activated cell culture solution was diluted in buffer solution (0.14 M sodium acetate), lysozyme solution (10 mg/mL) and crystallisation solution (10 mg/mL lysozyme with 80 mg/mL NaCl). The number of S. cerevisiae cells were determined by the spread plate technique. The Gram staining method was used to observe the status of S. cerevisiae, and the cells of S. cerevisiae were observed under the microscope (Olympus DP71 Japan). The FDA/PI staining kit was used to perform fluorescent staining on S. cerevisiae cells, and the staining results were observed under the microscope (Olympus DP71, Japan).

### Protein crystallisation

The sodium acetate buffer (0.14 M) was prepared by dissolving anhydrous sodium acetate in de-ionised water and followed by the addition of acetic acid to adjust the pH to 4.2. The sodium acetate buffer was filtered through a 0.22 µm filter. The preparation of protein solution was done by dissolving the protein (lysozyme, BSA or mixture) in the sodium acetate buffer. For example, to prepare a lysozyme solution with a target concentration of 20 mg/mL, lysozyme (20 mg) was dissolved in sodium acetate buffer (1 mL). Other solutions with concentrations of lysozyme from 10.0 to 2.5 mg/mL were prepared by dissolving corresponding amounts of lysozyme. Once lysozyme was fully dissolved, the solution was filtered through a 0.22 µm filter. Protein crystallization experiments were conducted using a conventional hanging-drop vapor diffusion technique. Crystallization drops were made by mixing 1.0 μL of protein solution and an equal volume of precipitation solution (80 mg/mL sodium chloride), with three different concentrations (0–5 × 10^6^ cfu/mL) of the S. cerevisiae, shown in Table 2. The final hanging drops of the mixture (2 μL) were sealed with a reservoir of sodium chloride solution (80 mg/mL), which were placed in the incubator at 20 °C $\pm$ 0.2 °C. In each crystallisation condition, 30–50 droplets were observed during the crystallization in the hanging droplets under a microscope (Olympus DP71) with time intervals between 1 and 24 h, dependent on the experimental conditions. To observe the aggregation of lysozyme during the crystallisation, hanging droplet experiments with fluorescent lysozyme at 20 mg/mL with low concentration of S. cerevisiae was performed as describe above, placing in the dark room for 4 h, then droplets were observed under an optical microscope (Olympus DP71) and fluorescence microscope (Olympus, Japan), with the green fluorescence at 495 nm.

**Table 2:** Experimental conditions of the crystallisation with and without biotemplates.

C_SC_ (cfu/mL)	C_NaCl_ (mg/mL)	C_Lys_ (mg/mL)	Observation interval (h)	Total time (h)
C_SC_ [Ref]: 0.0 ($\pm$ 5 × 10^5^)	80	2.5	6–24	96
C_SC_ [Low]: 5.0 × 10^4^ ($\pm$ 5 × 10^5^)		5.0	6–12	48
C_SC_ [High]: 5.0 × 10^6^ ($\pm$ 5 × 10^7^)		7.5	6–12	36
		10.0	6	24
		20.0	1	8

*C*_*SC*_ concentration of S. cerevisiae, *C*_*NaCl*_ concentration of sodium chloride, *C*_*Lys*_ concentration of lysozyme.

### Samples for SEM

The solution of hanging droplets with lysozyme crystals was contacted the silicon wafer carrier, then the filter paper was used to absorb the solution on the silicon wafer. The sample on the silicon wafer was air-dried and sprayed with Pt film, then was observed by scanning electron microscope (SEM, JEOL, Japan) .

## Supplementary Information

Supplementary Information.
