# Supplementary material for: Macromolecular protein crystallisation with biotemplate of live cells
Source: Sci Rep. 2022 Feb 22;12:3005. doi: 10.1038/s41598-022-06999-7 (PMC8864025; doi:10.1038/s41598-022-06999-7)
Supplement: Supplementary file 1 — Supplementary Information. [file 41598_2022_6999_MOESM1_ESM.docx]

**Macromolecular Protein Crystallisation with Biotemplate of Live Cells**

Mubai Sun^1^, Huaiyu Yang*^2^, Xinyu Miao^1^, Weixian Wang^3^, Jinghui Wang*^1^

1 Agricultural Products Processing Research Institute, Jilin Academy of Agricultural Science, Changchun, Jilin, 130124, China

2 Department of Chemical Engineering Loughborough University, Leicestershire, Le113rh, UK

3 School of Materials and Chemistry, University of Shanghai for Science and Technology, Shanghai, 200093, China

**Supporting information**

**
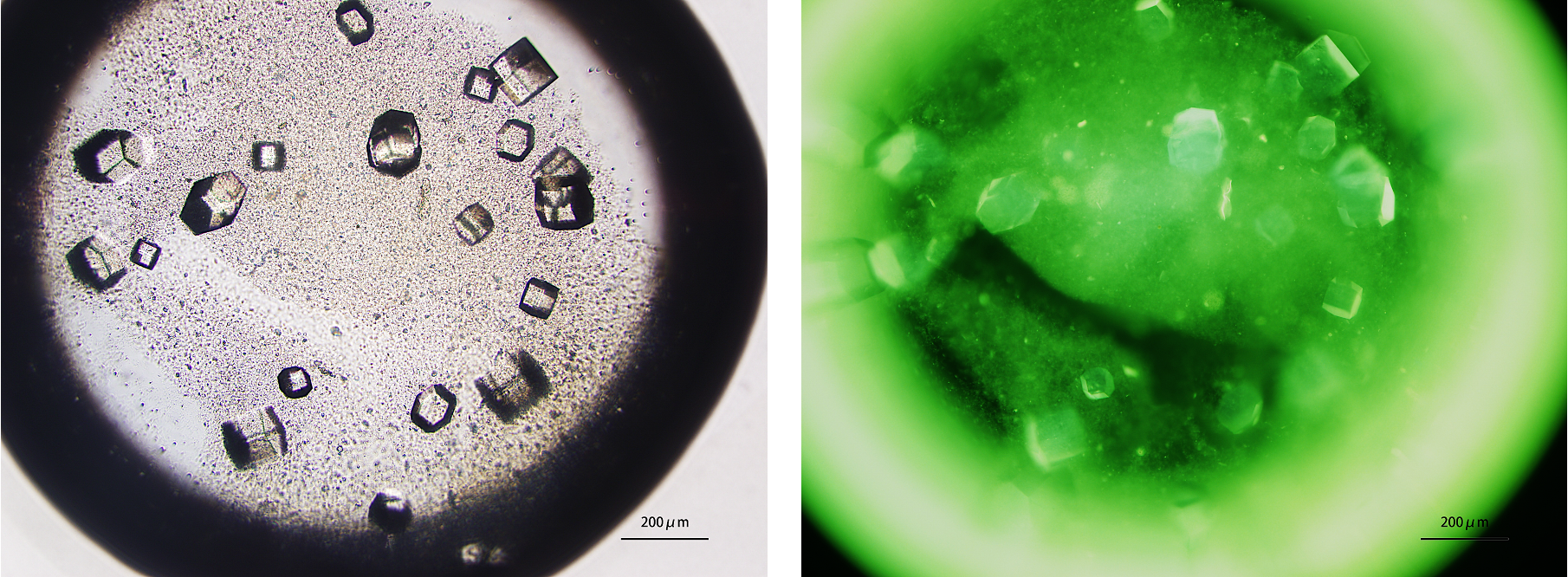
**

Figure S. Optical and fluorescence microscope images of the fluorescent lysozyme and crystals with the fluorescent lysozyme.

The tagged lysozyme molecules in the solution show fluorescence, as green colour all around the whole droplet under fluorescence microscope, but fluorescence of the tagged lysozyme disappears with the formation of the crystals of the tagged lysozyme, showing much the lighter colour / white colour, under the green colour background.
